# Health behavior of working-aged Finns predicts self-reported life satisfaction in a population-based 9-years follow-up

**DOI:** 10.1186/s12889-021-11796-4

**Published:** 2021-10-09

**Authors:** Säde Stenlund, Heli Koivumaa-Honkanen, Lauri Sillanmäki, Hanna Lagström, Päivi Rautava, Sakari Suominen

**Affiliations:** 1 Department of Public Health, University of Turku, 20014 Turku, Finland; 2 Research Services, Turku University Hospital, 20014 Turku, Finland; 3 Institute of Clinical Medicine (Psychiatry), University of Eastern Finland, 70211 Kuopio, Finland; 4 Mental health & Wellbeing Center, Kuopio University Hospital, 70029 Kuopio, Finland; 5 Department of Public Health, University of Helsinki, 00014 Helsinki, Finland; 6 Centre for Population Health Research, University of Turku and Turku University Hospital, 20014 Turku, Finland; 7 School of Health Sciences, University of Skövde, 54128 Skövde, Sweden

**Keywords:** Health behavior, Life satisfaction, Subjective well-being, Longitudinal study, Follow-up

## Abstract

**Background:**

Previous studies have shown positive association between health behavior and life satisfaction, but the studies have mostly been cross-sectional, had follow-up times up to 5 years or focused on only one health behavior domain. The aim of the study was to explore how principal health behavior domains predict life satisfaction as a composite score in a previously unexplored longitudinal setting.

**Methods:**

The present study tested whether a health behavior sum score (range 0–4) comprising of dietary habits, smoking, alcohol consumption, and physical activity predicted subsequent composite score of life satisfaction (range 4–20). Data included responses from 11,000 working-age Finns who participated in the Health and Social Support (HeSSup) prospective population-based postal survey.

**Results:**

Protective health behavior in 2003 predicted (*p* < .001) better life satisfaction 9 years later when sex, age, education, major diseases, and baseline life satisfaction were controlled for. The β in the linear regression model was − 0.24 (p < .001) corresponding to a difference of 0.96 points in life satisfaction between individuals having the best and worst health behavior.

**Conclusion:**

Good health behavior has a long-term beneficial impact on subsequent life satisfaction. This knowledge could strengthen the motivation for improvement of health behavior particularly on an individual level but also on a policy level.

**Supplementary Information:**

The online version contains supplementary material available at 10.1186/s12889-021-11796-4.

## Background

Health behavior is one of the presumed pathways in the well-established association between subjective well-being and in various ways determined good health [1, 2]. However, further understanding of this link is needed in longitudinal settings [3]. Subjective well-being refers to the extent to which a person feels that his or her life is going well [3] and life satisfaction represents the cognitive component of the personal evaluation [4].

The four major domains of modifiable health behavior with substantial impact on the risk of non-communicable diseases comprise of dietary habits, smoking, alcohol consumption, and physical activity [5, 6]. Thus far, the association of health promoting behavior with life satisfaction is well established in cross-sectional settings [7, 8], but few studies have focused on corresponding longitudinal associations. Healthy dietary patterns have been positively associated with good life satisfaction on a day-to-day basis [9, 10], have improved life satisfaction if changed in a favorable direction even when adjusted for other health behaviors [11, 12], and have predicted better life satisfaction in a 2-year follow-up [11]. Furthermore, being a smoker predicted poorer life satisfaction 4 years later [13], and high alcohol consumption poorer life satisfaction during a 15-year follow-up [14]. In addition, physical activity has shown a positive association with life satisfaction on a day-to-day basis [15] and increased physical activity has been shown to improve life satisfaction [16]. The effect has varied according to intensity of exercise, i.e. moderately intensive exercise improves life satisfaction, but vigorous does not [17]. Further, an overall additive effect has been observed, i.e. the more health behavior domains are on a favorable level, the stronger their association with life satisfaction [18].

Studies combining various principal domains of health behavior are encouraged [19] and for that purpose a sum score can be constructed (e.g. [20, 21]). A sum score has major advantages as health behaviors tend to cluster [19] and depicts the general situation of health behavior when co-occurrence is analyzed [22].

Previous studies have indicated several factors that can influence the results obtained. Age, sex, education, socioeconomic status, and health are the main covariates that have been repeatedly explored. Life satisfaction generally follows a U-shaped curve by age, the lowest life point being in the middle age [23, 24] and men generally show better life satisfaction than women [25]. Further, higher education generally results in better socioeconomic status that associates with better life satisfaction [26] but the effect of education does not disappear when adjusted for socioeconomic status [27]. Better health status (both objective and subjective) also associates with higher life satisfaction [2].

Due to the continuous increase of chronic diseases, innovative strategies are needed to decrease their burden in the individual and healthcare levels [5]. A better understanding of the link between health behavior and life satisfaction could enhance and support new and positive strategies for health behavior change. Thus far, however, research has mainly limited to single health behaviors and short follow-ups. As better health behavior leads to better health and prevents diseases, we hypothesized that it also predicts better life satisfaction [2]. Thus, we expect that a better health behavior score at baseline will result in a better subsequent life satisfaction score at follow-up. In a long follow-up, the effect could, however, be confounded by multiple changes happening in life. Adjusting for covariates, especially for baseline life satisfaction, assumably reduces the effect as they can correlate with baseline health behavior and are also determinants of subsequent life satisfaction.

The aim of the study was to explore how the four principal health behavior domains predict life satisfaction as a composite score in a previously unexplored longitudinal setting. Thus, we analyzed the predictive power of dietary habits, smoking, alcohol consumption, and physical activity as a composite measure in a 9-year prospective follow-up (in 2003–2012) in a large population-based and randomly selected sample of working-aged Finns.

## Methods

### Participants

Data for the present study originated from the longitudinal study on Health and Social Support (HeSSup), which was initiated in 1998. A random sample of age groups 20–24 years (group 1), 30–34 years (group 2), 40–44 years (group 3), and 50–54 years (group 4) of the Finnish working-age population (*n* = 64,797) was drawn from the national population register. Respondents gave their signed consent for a prospective follow-up including certain national health registry data. Details on the study population are given elsewhere [23].

**Fig. 1:**
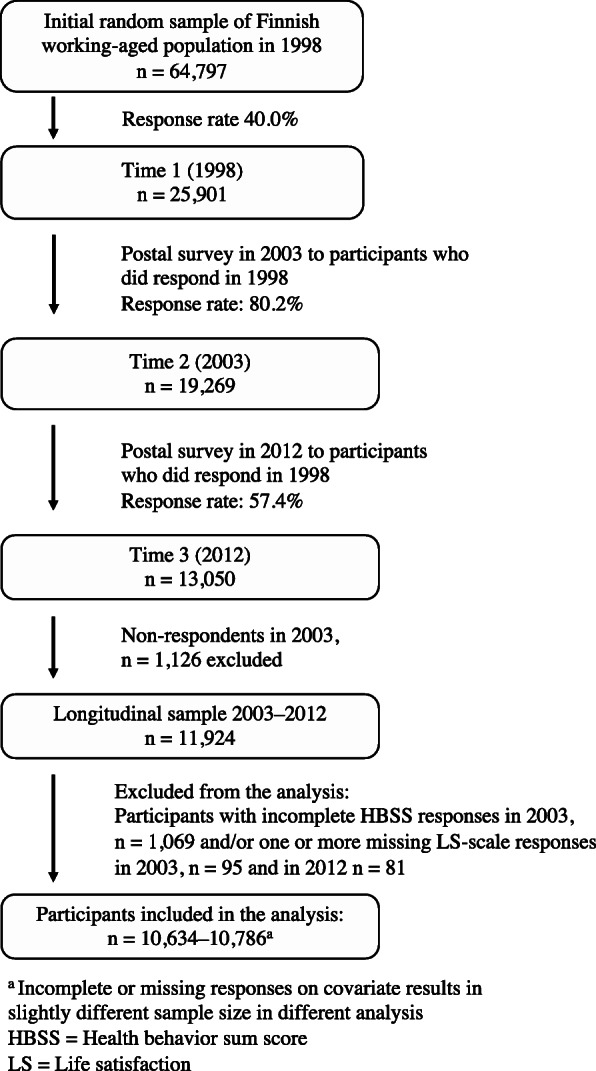
Inclusion and exclusion criteria for the present study sample. From: The Finnish population-based Health and Social Support Study

The first postal survey in 1998 (Time 1) had a response rate of 40.0% and resulted in data being collected from 25,901 individuals (Fig. 1). In 2003 (Time 2) and 2012 (Time 3), follow-up surveys were sent to all the individuals who responded in 1998. These surveys resulted in 19,269 responses (rate 80.2%) at Time 2 [24] and in 13,050 responses (rate 57.4%) at Time 3; the response rates being calculated from the Time 1 respondents. The three surveys comprised a number of unchanged items presented in an identical or almost identical order, but only the data from Time 2 and Time 3 included life satisfaction and dietary measures. Thus, the present 9-year follow-up study only included participants (*n* = 11,924) who have survey data from both Time 2 (in this study called baseline) and Time 3 (follow-up).

### Measures

The participants reported their habitual frequency of eating or drinking selected dietary components in a short non-validated food propensity questionnaire. *The dietary composite measure* was formed from ten items or groups: dark bread (≥ 2/day); pastries and sweets (≤ 1–2/week); fat free milk (≥ 1/day); sausages (≤ 1–2/week); red meat (≤ 1–2/week); chicken or turkey (≤1–2/week); fish (≥ 1–2/week); fresh fruits and berries (≥ 2/day); vegetables (≥ 2/day); alcohol use (women: < 70 g/week, men: < 140 g/week). The measure describes adherence to dietary recommendations in line with the Nordic Nutrition Recommendation 2004 [25]. Each recommended choice provided one point for the composite measure, so the overall score varied from 0 to 10, the maximum indicating perfect adherence to the recommendations [26]. For the analyses, we multiplied the score by 10 to have a percentage scale ranging from 0 to 100.

*Smoking* was dichotomized into current smokers and former smokers in combination with individuals who had never smoked regularly. *Alcohol consumption* was converted to grams per week. The cut-off point between heavy and moderate drinkers was 140 g/week for women and 280 g/week for men according to Finnish guidelines [27].

*Physical activity* was measured by intensity and time spent on leisure physical activity or physical activity related to commuting (hours in week) and was converted to a Metabolic Equivalent Task (MET). A MET value of 2 corresponds to approximately 30 min of walking per day and was the cut-off point for physically active individuals (c.f. below) [28].

Further, *a health behavior sum score* (HBSS, range 0–4) was calculated to represent the total number of protective health behaviors at baseline: dietary habits, smoking, alcohol consumption, and physical activity each scoring 0 (risky behavior) or 1 (protective behavior). Only participants who had reported all four health behavior domains were included in the analysis (*n* = 10,855, 83.2%). The cut-off values for the different domains were as follows:

1. Dietary patterns: sum score ≤ median (i.e. 0–50), HBSS = 0 vs. sum score > median, (i.e. 60–100), HBSS = 1.
2. Smoking: smokers, HBSS = 0 vs. former smokers / never being a regular smoker, HBSS = 1.
3. Alcohol consumption: heavy drinkers (women ≥140 g/week and men ≥280 g/week), HBSS = 0 vs. non-drinkers and moderate drinkers, HBSS = 1.
4. Physical activity: inactive MET < 2, HBSS = 0 vs. active MET ≥2, HBSS = 1.

*Life satisfaction* was measured with a four-item life satisfaction scale containing items of interest and happiness in life, ease of living, and feelings of loneliness. The items were scored as follows: very interesting/happy/easy/not at all lonely = 1; fairly interesting/happy/easy = 2; cannot say = 3; fairly boring/unhappy/hard/lonely = 4; very boring/unhappy/hard/lonely = 5. The range of the life satisfaction sum score was 4–20, a lower score indicating better life satisfaction [14, 29]. Allardt modified the four-item life satisfaction scale for welfare studies in the Nordic Countries [30] from quality of life studies [31, 32]. It has later been used in large population-based cohorts [29, 33, 34] and in several studies of somatic and psychiatric patient populations (e.g. [10, 35–38]). For 41 participants in 2003 and 54 in 2012 one response was missing and replaced by the mean of the remaining responses. If more than one item response was missing, the respondent was excluded from the analysis (*n* = 95 in 2003 and *n* = 81 in 2012).

*Baseline education and health* were included as potential confounders in the analyses as both are major factors affecting multiple areas of life (including health behavior and life satisfaction). Education was categorized into four groups: 1) no professional education; 2) vocational course/school/apprenticeship contract; 3) college; 4) university degree/polytechnic/higher. Health status was taken into account by self-reported diseases at baseline and categorized into the following three groups: 0, 1, ≥ 2 diseases. A translation of the questionnaire items used for the manuscript, including a list of the 35 diseases, is provided in the Additional file 1. The groups presumed to represent better outcomes in life satisfaction were chosen as reference: male, older, individuals having higher education, and fewer diseases.

### Statistical analysis

Descriptive statistics were calculated for the study population. The prevalence of each health behavior was reported for sex, age group, education, and diseases. A health behavior sum score at baseline (HBSS_2003_) was created as described above. The Cronbach’s alpha estimates for HBSS and life satisfaction score (LSscore) were computed. Several general linear models (GLM) with individual health behaviors as well as a health behavior sum score at baseline as independent variables and life satisfaction in the year 2012 (LSscore_2012_) as dependent variable were analyzed. Model 1 explored their crude association. In model 2, sex, age, education, and major diseases at baseline were added as covariates. To analyze the impact of life satisfaction at baseline, it was added to model 2 as a covariate to create model 3. The impact of the following interaction terms in respect for the baseline health behavior sum score (HBSS_2003_) were explored: *participant’s sex, *age, *education, and *diseases. None of these interactions was statistically significant. Data were analyzed with SAS software (version 9.4; SAS Institute Inc. Cary, NC, USA 2016).

## Results

In the study participants, there was a higher share of women (*n* = 6930, 63.8%) than men (*n* = 3925, 36.2%). In addition, the age group for 55–59 years in 2003 was slightly overrepresented (32.2%) compared to the younger age groups. The largest educational group (39.0%) among the participants had college-level education. Over half of the study population (58.8%) reported at least two major diseases.

The highest percentage of participants (42.4%) reported three favorable health behaviors, 26.1% reported all four favorable health behaviors, 24.5% reported two, 6.2% reported one, and 0.9% none. The mean LSscore after the follow up was 8.37 (SD 3.18). Women more often reported all health behavior domains as protective (HBSS_2003_ = 4) than men (30.9% vs. 17.7%). For details of the distribution of the study characteristics, see Table 1. The estimates for Cronbach’s alpha were as following: HBSS_2003_, 0.28; LSscore_2003_, 0.76; and LSscore_2012_ 0.76.

**Table 1:** Baseline characteristics and subsequent life satisfaction by subgroup. As percentages (numbers) unless otherwise stated. From: The Finnish population-based Health and Social Support Study

Variable	Share of the study population	Diet score 60–100	Non-smoking	Moderate or no alcohol consumption	Physically active (MET ≥2)	HBSS_2003_ = 0	HBSS_2003_ = 1	HBSS_2003_ = 2	HBSS_2003_ = 3	HBSS_2003_ = 4	LSscore_2003_^a^ (SD)	LSscore_2012_^a^ (SD)
Whole sample		39.1 (4245)	80.7 (8759)	94.8 (10,292)	72.2 (7832)	0.9 (93)	6.2 (670)	24.5 (2654)	42.4 (4602)	26.1 (2836)	8.53 (3.20)	8.37 (3.18)
Sex												
Male	36.2 (3925)	27.9 (1095)	77.7 (3048)	92.2 (3620)	68.4 (2686)	1.5 (58)	8.9 (348)	29.3 (1150)	42.7 (1675)	17.7 (694)	8.58 (3.18)	8.36 (3.16)
Female	63.8 (6930)	45.5 (3150)	82.4 (5711)	96.3 (6672)	74.3 (5146)	0.5 (35)	4.7 (322)	21.7 (1504)	42.2 (2927)	30.9 (2142)	8.51 (3.20)	8.38 (3.20)
Age (2003)												
25–29	20.6 (2234)	34.3 (766)	79.6 (1778)	96.9 (2164)	80.4 (1796)	0.5 (11)	4.7 (104)	23.1 (517)	46.6 (1042)	25.1 (560)	8.47 (3.15)	8.43 (3.15)
35–39	20.7 (2246)	34.6 (777)	78.7 (1768)	96.2 (2160)	72.9 (1637)	0.6 (14)	6.8 (153)	25.5 (572)	43.8 (983)	23.3 (524)	8.58 (3.28)	8.67 (3.38)
45–49	26.6 (2885)	39.7 (1146)	78.2 (2257)	92.8 (2677)	71.2 (2053)	1.3 (36)	7.6 (219)	24.9 (719)	40.5 (1168)	25.8 (743)	8.64 (3.31)	8.57 (3.31)
55–59	32.2 (3490)	44.6 (1556)	84.7 (2956)	94.3 (3291)	67.2 (2346)	0.9 (32)	5.6 (194)	24.2 (846)	40.4 (1409)	28.9 (1009)	8.45 (3.06)	7.96 (2.90)
Education (2003)												
No professional education	12.0 (1302)	31.9 (415)	73.4 (955)	93.6 (1219)	67.7 (881)	1.5 (19)	8.9 (116)	30.3 (394)	40.4 (526)	19.0 (247)	8.89 (3.33)	8.58 (3.26)
Vocational school	29.0 (3136)	33.5 (1051)	74.9 (2350)	94.3 (2956)	68.2 (2138)	1.0 (31)	9.0 (281)	28.0 (876)	42.4 (1330)	19.7 (618)	8.76 (3.30)	8.54 (3.27)
College	39.0 (4218)	41.2 (1738)	81.6 (3443)	95.2 (4014)	73.3 (3090)	0.8 (35)	5.2 (219)	23.6 (996)	42.6 (1798)	27.7 (1170)	8.40 (3.15)	8.24 (3.07)
University	19.9 (2154)	47.6 (1026)	91.6 (1973)	95.6 (2059)	78.6 (1694)	0.4 (8)	2.4 (51)	17.6 (378)	42.9 (923)	36.9 (794)	8.24 (3.01)	8.08 (3.01)
Diseases (2003)												
0	17.9 (1931)	38.0 (733)	83.6 (1615)	96.0 (1853)	76.8 (1483)	0.5 (10)	4.6 (89)	22.7 (438)	44.4 (857)	27.8 (537)	7.84 (2.73)	7.91 (2.85)
1	23.3 (2522)	37.5 (945)	81.8 (2064)	95.8 (2417)	73.6 (1860)	0.6 (15)	6.0 (151)	23.6 (596)	43.5 (1097)	26.3 (663)	8.12 (2.94)	7.98 (2.89)
2 or more	58.8 (6355)	40.1 (2545)	79.4 (5043)	94.1 (5978)	70.1 (4456)	1.1 (68)	6.7 (428)	25.3 (1607)	41.4 (2628)	25.6 (1624)	8.91 (3.37)	8.60 (3.31)

^a^ LSscore = Life satisfaction score; range 4–20, lower scores indicates better life satisfaction

HBSS_2003_ = Health behavior sum score i.e. number of protective health behaviors at baseline in 2003

Diseases (2003) = Number of diseases participants reported at baseline in 2003. List of diseases is found in the Additional file 2

The size of subgroups varies slightly depending on missing values

The protective behavior of the four studied behavior domains predicted significantly better life satisfaction after follow-up (*p* < .001) in all the linear models (Table 2). In the crude model 1, the β estimate for subsequent LSscore was − 0.51. Thus, each protective health behavior changed the LSscore towards better life satisfaction by 0.51 points, resulting in a total of 2.04 points for those with all favorable health behaviors. In model 2 with sex, age, education, and major diseases as covariates, the β decreased to − 0.47. In model 3, also adding the baseline life satisfaction in 2003 in model 2, reduced the β to − 0.24 (*p* < .001), resulting in a total of 0.96 points better life satisfaction after follow-up for those with all protective health behaviors compared to those with none.

**Table 2:** Details of models where health behavior sum score predicts life satisfaction after 9 years. Non-standardized estimates for linear regression models in which the baseline health behavior sum score predicts life satisfaction^a^. From: The Finnish population-based Health and Social Support Study

Model	Estimate	Standard error	*p*-value	AIC	R^2^
Model 1: Crude linear model where health behavior sum score in 2003 predicts LSscore_2012_, no covariates	− 0.51	0.033	< .001	55,163	0.02
Model 2: Model 1 + sex, age, education, diseases as covariates	− 0.47	0.034	< .001	54,490	0.04
Model 3: Model 2 + LSscore_2003_ as a covariate	−0.24	0.031	< .001	51,826	0.23

LSscore_2012_ = Life satisfaction score after the 9-year follow-up in 2012; range 4–20, lower scores indicate better life satisfaction

LSscore_2003_ = Life satisfaction score at baseline in 2003; range 4–20, lower scores indicate better life satisfaction

^a^lower scores indicate better life satisfaction

The details of the estimates of model 3 are presented in Table 3. Baseline life satisfaction increased life satisfaction after follow-up by a β estimate of 0.44 (*p* < .001) i.e. one point lower life satisfaction score in 2003 indicated a decrease of 0.44 in the life satisfaction score after follow-up. Age (p < .001), education (*p* = .02), and diseases (p < .001) were statistically significant covariates. Belonging to the age group 2, having lower education, and having more than two diseases was associated with poorer life satisfaction and a smaller probability of having all protective health behaviors (see also Table 1). Sex was not a significant covariate.

**Table 3:** Estimates for health behavior sum score predicting subsequent life satisfaction adjusted for covariates. Non-standardized estimates for the linear regression model in which the baseline health behavior sum score predicts subsequent life satisfaction^a^. From: The Finnish population-based Health and Social Support Study

	Estimate	Standard error	*p*-value
Intercept	4.65	0.17	< .001
HBSS_2003_	−0.24	0.031	< .001
LSscore_2003_	0.44	0.0086	< .001
Sex			
Male	Reference		
Female	0.069	0.057	.23
Age (2003)			
25–29	0.56	0.080	< .001
35–39	0.70	0.078	< .001
45–49	0.50	0.071	< .001
55–59	Reference		
Education (2003)			
No professional education	0.18	0.10	.065
Vocational school	0.17	0.080	.03
College	0.00028	0.074	.99
University or higher	Reference		
Diseases (2003)			
0	Reference		
1	−0.026	0.084	.76
2 or more	0.35	0.075	< .001

LSscore_2003_ = Life satisfaction score in 2003, lower scores indicate better life satisfaction

HBSS_2003_ = Health behavior sum score i.e.number of protective health behaviors at baseline in 2003

^a^lower scores indicate better life satisfaction

When studying linear regression models for dichotomized individual health behaviors all showed significant (p < .001) predictive power and similar Akaike Information Criterion (AIC) where alcohol consumption had the highest estimate for prediction as follows: alcohol consumption 0.62, smoking status 0.38, physical activity 0.26, and dietary habits 0.19. Details of the models on individual health behaviors are provided in Additional file 2, Tables 2-5.

## Discussion

The present 9-year follow-up study of a population of 11,000 working-aged Finns found that health behavior predicted life satisfaction even after controlling for sex, age, baseline education, major diseases, and baseline life satisfaction. It is, to our knowledge, the first study to assess the predictive power of four major domains of modifiable health behavior as a sum score on subsequent life satisfaction. However, the strongest predictor was baseline life satisfaction among the covariates 9-years previously, which halved, but did not eradicate the significant impact of health behavior on subsequent life satisfaction. Alcohol consumption and smoking showed stronger effect on life satisfaction compared to physical activity and dietary habits.

Our study contributes significantly to research on multiple health behaviors and their impact on supporting or creating good life satisfaction. Life satisfaction can here be regarded as also representing more generally good mental health. However, it is obvious that for most people over the course of time, life circumstances, health behavior, and factors influencing life satisfaction vary substantially. Therefore, it is remarkable that a statistically significant longitudinal association between health behavior and life satisfaction persisted in a 9-year follow-up. Previously, heavy alcohol consumption predicted poorer life satisfaction during a 15-year follow-up with the same, but categorized, life satisfaction scale as used here [14]. Another study [13] concluded by structural equation modeling that smoking predicted significantly poorer life satisfaction 4 years later. Further, the study by Mujcic and Oswald [11] explored the association between initial fruit and vegetable consumption and later life satisfaction in a 2-year follow-up adjusting for exercise, smoking, alcohol consumption and eating patterns; consumption of fruit and vegetables predicted subsequent life satisfaction and an average increase of 0.24 in life satisfaction (range: 0–10) was observed when the consumption of fruits and vegetables was increased by eight portions a day. The study by Ocean et al. [12] concluded that a change in fruit and vegetable consumption resulted in better well-being 5 years later when adjusted for smoking status, walking 10 min per day and longstanding health condition. In line with earlier research [23, 24], a U-shaped curve was observed in life satisfaction by age where the oldest age group showed best life satisfaction. Furthermore, lower life satisfaction was observed in those having two or more diseases, but not having one, when compared to no diseases, which is in line with earlier research showing better health resulting in better life satisfaction [2]. Higher education was also associated with better life satisfaction as in earlier research [26], but not consistently when comparing no education or college level education to university level education. In contrast to earlier research [25], no difference between sexes was observed which might be due to the sex equality in Finland. None of the covariates showed significant interaction in how health behavior predicts life satisfaction suggesting that all covariate groups benefit similarly of good health behavior in respect to life satisfaction.

In a non-response analysis of the Time 1 study population [39], externally caused mortality of the non-respondents was found to be significantly higher for men whereas disease mortality was significantly higher for women as compared to respondents in the 7-year follow-up, although the absolute differences were small. In another earlier non-response analysis of the present data, the respondents in Time 1 showed satisfactory representativeness to national morbidity statistics of the corresponding Finnish population, although women, younger people, those in employment, and individuals with a higher education were somewhat over-represented. On the other hand, smoking and the use of anti-depressants were less prevalent than among the corresponding Finnish population [23]. Compared to Time 1, the present study population showed a higher proportion of women (63.8% vs. 59.2% in 1998) and the proportion of those with high education was lower (19.9% vs. 33.8% in 1998). In 2003, the oldest age group represented the highest proportion of respondents (32.2%) whereas in 1998 the four age groups were fairly equally represented. Therefore, even though the Time 1 study population was mostly fairly representative of the general population, attrition could also be responsible for some changes. However, the covariate groups showed a satisfactory distribution and thus the results can be generalized to the working-aged Finnish population.

To the best of our knowledge, the present study is the first to identify a long-term predictive effect of a sum score comprising multiple health behavior domains to life satisfaction in the future. The observed difference in subsequent life satisfaction between individuals reporting four protective health behaviors in comparison to those with none was almost a third of the magnitude of the standard deviation of the outcome. Furthermore, the difference in life satisfaction related to baseline health behavior reached a twofold level as compared to the corresponding difference between those having a baseline university education and those lacking any professional education. Since good health behavior improves health, better health is presumably one of the routes by which health behavior can influence life satisfaction [3].

The results of the present study may have many implications for clinical medicine, health policy, and health promotion. Innovative solutions for health promotion are needed as the increasing prevalence of chronic non-communicable diseases in combination with advanced but increasingly expensive medical technology challenges healthcare around the world [5]. As focusing on the long-term risk perspective alone is generally perceived by most people as arduous, our results could provide a more beneficial approach by emphasis on future mental well-being (i.e. life satisfaction and happiness) [4, 40] that can be supported by good health behavior. Moreover, improved health behavior reduces the costs of healthcare by prevention of diseases. It can also increase individual productivity via enhanced life satisfaction [41]. From an individual’s perspective, pursuing happiness and life satisfaction is of central importance. Recognizing the role of health behavior in improving one’s life satisfaction could strengthen motivation for making changes for the better. In addition, the results of the present study emphasize that improvement in one health behavior domain might already exert a protective impact. Thus, improving health behavior should not necessarily focus concomitantly on all domains to enable a favorable development in life satisfaction.

### Strengths and limitations

The present study has several strengths. The data enabled evaluation of the longitudinal association between multiple health behaviors and subsequent life satisfaction which has been lacking in previous research. The results are robust due to the large population-based sample, consistent survey procedure, and a long follow-up time. Both of its two indicators (the health behavior sum score and the life satisfaction scale) are composite measures covering health behavior and life satisfaction in broader terms than commonly applied in research. The health behavior sum score gives an evaluation of the co-occurrence of multiple positive health behaviors, not of only one health behavior domain at a time. Each domain was dichotomized into either risky or protective behavior as in other studies where multiple health behaviors have been in focus [22]. No consensus as regards cut-off points [22] or weighting of the different areas has been established [19]. We mostly utilized cut-off points that have been used in previous studies of this data, but in the case of the diet score, due to missing information, a median was used.

The Cronbach’s alpha for the health behavior sum score is relatively low, indicating that the behaviors are not consistently co-varying. Therefore, a sum score of the dichotomized variables for four major health behaviors is not necessarily the best scale to reflect health behavior but was used in the present study to include the health behaviors of major public health concern. Further studies on health behavior scales are needed, as the study of multiple health behaviors is encouraged [19]. The outcome was measured with the 4-item life satisfaction scale that takes into account the cognitive, affective, and social dimensions of well-being. Previously, this has been reported as being quite stable at an individual level [e.g. 10]. Since an individual’s initial level of life satisfaction predicts subsequent life satisfaction, this effect was controlled for in the analysis of the present study.

The study also has limitations. Objective assessment of health behavior was beyond the scope of this large prospective population-based survey. Thus, health behavior was based on self-reports, which can result in some bias. In contrast, life satisfaction as an indicator of subjective well-being is most frequently studied by self-reports [3]. As a result of dichotomizing the health behavior measures it was impossible to evaluate small improvement in health behavior. Alcohol consumption had double impact on the health behavior sum score; first it represented one of the four principal health behaviors in the sum score and additionally it was taken into account as one of the ten factors of the dietary composite measure. In the dietary measure alcohol-consumption reflected its effect on a balanced diet and the cut-off point was half of the risk-use level. Thus, the impact of alcohol consumption can be considered to be somewhat overrepresented in the health behavior sum score. However, we chose to include it in the dietary composite measure in order to include its effect as part of a balanced diet. Lastly, socioeconomic status [42] and employment status [43] affect life satisfaction and could be seen as appropriate covariates. On the other hand, education correlates positively with socioeconomic status and occupational class and hence, could roughly reflect their effect [44], too.

### Further research

A focus by future research on the mechanisms of changes in health behavior and life satisfaction would be especially beneficial as this knowledge is of importance to broaden the possibilities for early intervention. Including additional domains of health behaviors (e.g. sleep, sedentary time, meditation, screen time) in a composite measure would help to encounter future public health challenges. The association between health behavior and life satisfaction is assumably bidirectional and should be studied in the future.

## Conclusion

Good health behavior is protective to subsequent life satisfaction. This knowledge could strengthen the motivation for improvement of health behavior particularly on an individual level but also on a policy level.

## Supplementary Information

**Additional file 1. **Original questionnaire items**.** Non-validated translation of original questionnaire items in the Health and Social Support (HeSSup) survey. Total number of items in the survey was 103 in 2003 (112 in 2011) of which 12 were used for the manuscript and are presented here.

**Additional file 2.** Statistical details. Additional details on statistical models and detailed information about reported diseases.

## Data Availability

The dataset analyzed during the current study is not publicly available due to study data containing variables of personal and sensitive nature and hence, due to the present legislation of Finland and the General Data Protection Regulation (GDPR) of the European Union, cannot be made openly accessible inside or outside Finland. However, in some special cases data is available from the authors on reasonable request.

## Abbreviations

HeSSup study: Health and Social Support study
HBSS: Health Behavior Sum Score
LS: Life Satisfaction
LSscore: Life Satisfaction score
MET: Metabolic Equivalent Task
