# Supplementary material for: Health behavior of working-aged Finns predicts self-reported life satisfaction in a population-based 9-years follow-up
Source: BMC Public Health. 2021 Oct 9;21:1815. doi: 10.1186/s12889-021-11796-4 (PMC8501556; doi:10.1186/s12889-021-11796-4)
Supplement: Supplementary file 1 — Additional file 1. Original questionnaire items. Non-validated translation of original questionnaire items in the Health and Social Support (HeSSup) survey. Total number of items in the survey was 103 in 2003 (112 in 2011) of which 12 were used for the manuscript and are presented here. [file 12889_2021_11796_MOESM1_ESM.pdf]

Additional file 1

Original questionnaire items

A non-validated translation to English of the original questionnaire items in the Health and Social Support (HeSSup) survey. Total number of items in the survey was 103 in 2003 (112 in 2011) of which 12 were used for the manuscript and are presented here. Placement in the survey in 2003 is indicated by the item number before every question and in parenthesis for the year 2011. References are given where translations are obtained from previous publications. The question regarding physical exercise is not an exact translation but corresponds more to the tone by which this topic would be inquired in an English speaking population. Information about birth year was derived from the official personal identification code.

2 (2) What is your vocational education (choose one) [1]

☐ No professional training	☐ Vocational school	☐ Degree from university
☐ Vocational course (minimum length 4 months)	☐ College	
☐ Apprenticeship contract	☐ Polytechnic	

28 (28) During the past year, how much time in a week did you spend on physical activity in leisure time or while commuting? How intense has the physical activity been? (adapted from: 2,3)

	Amount of time spent on physical activity in a week				
Intensity of physical activity (answer each section below)	Not at all	Less than 0.5 hour	One hour	2–3 hours	4 hours or more
Comparable to walking	☐	☐	☐	☐	☐
Comparable to brisk walking	☐	☐	☐	☐	☐
Comparable to jogging	☐	☐	☐	☐	☐
Comparable to running	☐	☐	☐	☐	☐

32 (31)

How often have you consumed the following food items? [4]

	2 times a day or more often	Once a day	On 3–6 days a week	On 4–10 days a month	On 1–2 days a month	Rarely	Never
Rye bread[a]	☐	☐	☐	☐	☐	☐	☐
White bread	☐	☐	☐	☐	☐	☐	☐
Brown bread	☐	☐	☐	☐	☐	☐	☐
Pastry or bisquits[a]	☐	☐	☐	☐	☐	☐	☐
Potato chips or equivalent	☐	☐	☐	☐	☐	☐	☐
Sweets[a]	☐	☐	☐	☐	☐	☐	☐
Fresh fruit[a]	☐	☐	☐	☐	☐	☐	☐
Fresh vegetables[a]	☐	☐	☐	☐	☐	☐	☐
Berries[a]	☐	☐	☐	☐	☐	☐	☐

Mushrooms	☐	☐	☐	☐	☐	☐	☐
Cheese	☐	☐	☐	☐	☐	☐	☐
Fat free milk[a]	☐	☐	☐	☐	☐	☐	☐
Other milk	☐	☐	☐	☐	☐	☐	☐
Soured-milk products	☐	☐	☐	☐	☐	☐	☐
Tea	☐	☐	☐	☐	☐	☐	☐
Sausages[a]	☐	☐	☐	☐	☐	☐	☐
Beef[a]	☐	☐	☐	☐	☐	☐	☐
Pork[a]	☐	☐	☐	☐	☐	☐	☐
Chicken or turkey[a]	☐	☐	☐	☐	☐	☐	☐
Lamb[a]	☐	☐	☐	☐	☐	☐	☐
Salmon, rainbow trout or tuna[a]	☐	☐	☐	☐	☐	☐	☐
Other fish[a]	☐	☐	☐	☐	☐	☐	☐
Eggs	☐	☐	☐	☐	☐	☐	☐

[a] Items used in the diet score. [4]

54 (54) Has a doctor ever said, that you have or had had any of the following conditions (Indicate even, if your answer is no):

	No	Yes
Long-term bronchitis or bronchiectasis	☐	☐
Lung asthma	☐	☐
Allergic rhinitis e.g. hay fever	☐	☐
High blood pressure	☐	☐
Hypertension	☐	☐
High cholesterol	☐	☐
Diabetes	☐	☐
Myocardial infarction or coronary thrombosis	☐	☐
Angina pectoris, i.e. chest pain caused by coronary artery disease	☐	☐
Atrial fibrillation or atrial flutter	☐	☐
Stroke	☐	☐
Other cerebrovascular accident	☐	☐
Peptic ulcer	☐	☐
Celiac disease	☐	☐
Liver disease	☐	☐
Kidney disease	☐	☐
Rheumatoid arthritis	☐	☐
Arthrosis	☐	☐
Sciatica	☐	☐
Fibromyalgia	☐	☐
Cataract or glaucoma	☐	☐
Migraine	☐	☐
Epilepsy	☐	☐
Brain injury	☐	☐
Meningitis or encephalitis	☐	☐
Other cerebral disease of neurological disease	☐	☐
Depression	☐	☐
Panic disorder	☐	☐
Eating disorder	☐	☐
Other mental disorder	☐	☐
Malignant tumor	☐	☐
Other chronic or severe disease, which?	☐	☐

72 (72) How many cigarettes on average do you currently smoke daily? [1]

☐ None	☐ 15–19 cigarettes
☐ Less than 5 cigarettes	☐ 20–24 cigarettes
☐ 5–9 cigarettes	☐ 25–40 cigarettes
☐ 10–14 cigarettes	☐ over 40 cigarettes

How much do you drink the following alcoholic beverages on average? [5]

78 (80) Beer IN A WEEK	☐ Not at all<br>☐ Less than one bottle (0.33l)<br>☐ 1–4 bottles<br>☐ 5–12 bottler	☐ 13–24 bottles<br>☐ 25–47 bottles<br>☐ Over 48 bottles
79 (81) Wine or other equivalent mild alcoholic beverages IN A WEEK	☐ Not at all<br>☐ Less than a glass<br>☐ 1–4 glasses<br>☐ 1–2.5 bottles	☐ 3–4.5 bottles<br>☐ 5–9 bottles<br>☐ Over 10 bottles
80 (83) Spirits IN A MONTH	☐ Not at all<br>☐ Less than half a bottle (0.5l)<br>☐ 0.5–1.5 bottles<br>☐ 2–3.5 bottles	☐ 4–9 bottles<br>☐ 10–19 bottles<br>☐ Over 20 bottles

98 (102) Do you feel that your life at present is [6]

☐ Very interesting
☐ Fairly interesting
☐ Cannot say
☐ Fairly boring
☐ Very boring

99 (103) Do you feel that your life at present is [6]

☐ Very happy
☐ Fairly Happy
☐ Cannot say
☐ Fairly unhappy
☐ Very unhappy

100 (104) Do you feel that your life at present is [6]

☐ Very easy
☐ Fairly easy
☐ Cannot say
☐ Fairly hard
☐ Very hard

101 (105) Do you feel that at the present moment you are [6]

☐ Very lonely
☐ Fairly lonely
☐ Not at all lonely
☐ Cannot say

Note that as in earlier publications [6] the item of loneliness is a four level item but was stretched over a scale of 1–5 excluding value 2 to have the same range as the other responses. In addition, the item was reversed having the same direction of positive outcome i.e. not being lonely as the other items.

References:

1. Paljärvi T. Subjective measures of binge drinking and adverse health outcomes. University of Helsinki; 2014.
2. Fransson EI, Heikkilä K, Nyberg ST, Zins M, Westerlund H, Westerholm P, et al. Job strain as a risk factor for leisure-time physical inactivity: An individual-participant meta-analysis of up to 170,000 men and women. Am J Epidemiol. 2012;176:1078–89.
3. Virtanen P, Vahtera J, Broms U, Sillanmä Ki L, Kivimä Ki M, Koskenvuo M. Employment trajectory as determinant of change in health-related lifestyle: the prospective HeSSup study. Eur J Public Health. 2008;18:504–8.
4. Lagström H, Halonen JI, Kawachi I, Stenholm S, Pentti J, Suominen S, et al. Neighborhood socioeconomic status and adherence to dietary recommendations among Finnish adults: A retrospective follow-up study. Heal Place. 2019;55:43–50.
5. Paljärvi T, Mäkelä P, Poikolainen K, Suominen S, Car J, Koskenvuo M. Subjective measures of binge drinking and alcohol-specific adverse health outcomes: A prospective cohort study. Addiction. 2012;107:323–30.
6. Koivumaa-Honkanen H, Honkanen R, Viinamäki H, Heikkilä K, Kaprio J, Koskenvuo M. Self-reported Life Satisfaction and 20-Year Mortality in Healthy Finnish Adults. 2000.
