# Supplementary material for: Health behavior of working-aged Finns predicts self-reported life satisfaction in a population-based 9-years follow-up
Source: BMC Public Health. 2021 Oct 9;21:1815. doi: 10.1186/s12889-021-11796-4 (PMC8501556; doi:10.1186/s12889-021-11796-4)
Supplement: Supplementary file 2 — Additional file 2. Statistical details. Additional details on statistical models and detailed information about reported diseases. [file 12889_2021_11796_MOESM2_ESM.pdf]

Additional file 2
Statistical details

Table S1 Non-standardized estimates for linear regression model 2 in which the baseline health behavior sum score predicts subsequent life satisfaction[a] adjusted for sex, age, education and diseases but not for baseline life satisfaction. The Finnish population-based Health and Social Support Study.

		Estimate	Standard error	p-value
Intercept		8.65	0.16	< .001
$HBSS_{2003}$		–0.47	0.034	< .001
Sex	Male	Reference		
	Female	–0.10	0.064	.11
Age (2003)	25–29	0.75	0.089	< .001
	35–39	0.86	0.087	< .001
	45–49	0.65	0.080	< .001
	55–59	Reference		
Education (2003)	No professional education	0.32	0.092	.003
	Vocational school	0.24	0.089	.006
	College	0.0020	0.083	.98
	University or higher	Reference		
Diseases (2003)	0	Reference		
	1	0.065	0.094	.49
	2 or more	0.79	0.083	< .001

[a] $LSscore_{2012}$ = Life satisfaction score in 2012, lower scores indicate better life satisfaction

$HBSS_{2003}$ = Health behavior sum score i.e.number of protective health behaviors at baseline in 2003

Table S2 Non-standardized estimates for the linear regression model in which dichotomized baseline dietary habits predicts subsequent life satisfaction[a]. Observed statistical significance level of the covariates. The Finnish population-based Health and Social Support Study.

Model	AIC	Intercept (p-value)	Dietary habits (p-value)	$LSscore_{2003}$[a] (p-value)	Age	Sex	Education	Diseases
Model 1: Crude linear model where dietary habits 2003 predicts life satisfaction[a] score 2012, no covariates	55,330	8.05 (< .001)	-0.50 (< .001)	-	-	-	-	-
Model 2: Model 1 + sex, age, education, diseases as covariates	54,631	8.08 (< .001)	-0.43 (< .001)	-	< .001	.63	< .001	< .001
Model 3: Model 2 + $LSscore_{2003}$ as a covariate	51,873	4.29 (< .001)	-0.19 (< .001)	0.45 (< .001)	< .001	.62	.002	< .001

[a] $LSscore_{2012}$= Life satisfaction score in 2012; lower score indicates better life satisfaction.
$LSscore_{2003}$ = Life satisfaction score at baseline in 2003; lower scores indicate better life satisfaction

Table S3 Non-standardized estimates for the linear regression model in which a dichotomized baseline smoking status predicts subsequent life satisfaction[a]. Observed statistical significance level of the covariates. The Finnish population-based Health and Social Support Study.

Model	AIC	Intercept (p-value)	Smoking status (p-value)	$LSscore_{2003}$[a] (p-value)	Age	Sex	Education	Diseases
Model 1: Crude linear model where smoking status 2003 predicts life satisfaction[a] score 2012, no covariates	55,256	8.18 (< .001)	-0.91 (< .001)	-	-	-	-	-
Model 2: Model 1 + sex, age, education, diseases as covariates	54,583	8.17 (< .001)	-0.75 (< .001)	-	< .001	.87	< .001	< .001
Model 3: Model 2 + $LSscore_{2003}$ as a covariate	51,855	4.34 (< .001)	-0.38 (< .001)	0.45 (< .001)	< .001	.83	.007	< .001

[a] $LSscore_{2012}$= Life satisfaction score in 2012; lower score indicates better life satisfaction.
$LSscore_{2003}$ = Life satisfaction score at baseline in 2003; lower scores indicate better life satisfaction

Table S4 Non-standardized estimates for the linear regression model in which dichotomized baseline alcohol consumption predicts subsequent life satisfaction[a]. Observed statistical significance level of the covariates. The Finnish population-based Health and Social Support Study.

Model	AIC	Intercept	Alcohol consumption (p-value)	$LSscore_{2003}$[a] (p-value)	Age	Sex	Education	Diseases
Model 1: Crude linear model where alcohol consumption 2003 predicts life satisfaction[a] score 2012, no covariates	55,320	8.29 (< .001)	-1.17 (< .001)	-	-	-	-	-
Model 2: Model 1 + sex, age, education, diseases as covariates	54,609	8.25 (< .001)	-1.11 (< .001)	-	< .001	.94	< .001	< .001
Model 3: Model 2 + $LSscore_{2003}$ as a covariate	51,858	4.38 (< .001)	-0.62 (< .001)	0.45 (< .001)	< .001	.76	< .001	< .001

[a] $LSscore_{2012}$= Life satisfaction score in 2012; lower score indicates better life satisfaction.

$LSscore_{2003}$ = Life satisfaction score at baseline in 2003; lower scores indicate better life satisfaction

Table S5 Non-standardized estimates for the linear regression model in which dichotomized baseline physical activity predicts subsequent life satisfaction[a]. Observed statistical significance level of the covariates. The Finnish population-based Health and Social Support Study.

Model	AIC	Intercept	Physical activity (p-value)	$LSscore_{2003}$[a] (p-value)	Age	Sex	Education	Diseases
Model 1: Crude linear model where physical activity 2003 predicts life satisfaction[a] score 2012, no covariates	55,343	8.22 (< .001)	-0.49 (< .001)	-	-	-	-	-
Model 2: Model 1 + sex, age, education, diseases as covariates	54,626	8.15 (< .001)	-0.48 (< .001)	-	< .001	.71	< .001	< .001
Model 3: Model 2 + $LSscore_{2003}$ as a covariate	51,867	4.31 (< .001)	-0.26 (< .001)	0.45 (< .001)	< .001	.91	< .001	< .001

[a] $LSscore_{2012}$= Life satisfaction score in 2012; lower score indicates better life satisfaction.

$LSscore_{2003}$ = Life satisfaction score at baseline in 2003; lower scores indicate better life satisfaction

Table S6 Number of major diseases reported by the participants

The Finnish population-based Health and Social Support Study.

Number of Diseases	Frequency	Percentage
0	2136	18.01
1	2768	23.34
2	2399	20.23
3	1720	14.51
4	1170	9.87
5	634	5.35
6	428	3.61
7	230	1.94
8	150	1.27
9	93	0.78
10 or more	129	1.09

Table S7 Number of participants reporting specific diseases. The Finnish population-based Health and Social Support Study.

Number of Diseases	Frequency	Percentage
Long-term bronchitis or bronchiectasis	1065	9.1
Lung asthma	794	6.8
Allergic rhinitis e.g. hay fever	3517	29.9
High blood pressure	3834	32.5
Hypertension	1126	9.6
High cholesterol	2917	24.7
Diabetes	381	3.2
Myocardial infarction or coronary thrombosis	134	1.1
Angina pectoris, i.e. chest pain caused by coronary artery disease	232	2.0
Atrial fibrillation or atrial flutter	268	2.3
Stroke	76	0.7
Other cerebrovascular accident	251	2.1
Peptic ulcer	724	6.2
Celiac disease	179	1.5
Liver disease	179	1.5
Kidney disease	249	2.1
Rheumatoid arthritis	275	2.3
Arthrosis	1499	12.8
Sciatica	2183	18.6
Fibromyalgia	320	2.7
Cataract or glaucoma	280	2.4
Migraine	2514	21.3
Epilepsy	162	1.4
Brain injury	123	1.1
Meningitis or encephalitis	252	2.1
Other cerebral disease of neurological disease	274	2.3
Depression	1692	14.4
Panic disorder	731	6.2
Eating disorder	205	1.7
Other mental disorder	436	3.7
Malignant tumor	342	2.9
Other chronic or severe disease, which?	1618	14.1
